# Determination of the association of *GHR*/*AluI* gene polymorphisms with milk yield traits in Holstein and Jersey cattle raised in Turkey

**DOI:** 10.5194/aab-64-417-2021

**Published:** 2021-09-23

**Authors:** Ozden Cobanoglu, Ertugrul Kul, Eser K. Gurcan, Samet H. Abaci, Soner Cankaya

**Affiliations:** 1 Department of Genetics, Faculty of Veterinary Medicine, Bursa Uludag University, 16059, Bursa, Turkey; 2 Department of Animal Science, Faculty of Agriculture, Kirsehir Ahi Evran University, 40200, Kirsehir, Turkey; 3 Department of Animal Science, Faculty of Agriculture, University of Namik Kemal, 59030, Tekirdag, Turkey; 4 Department of Animal Science, Faculty of Agriculture, University of Ondokuz Mayis, 55139, Samsun, Turkey; 5 Department of Sport Management, Faculty of Yaşar Doğu Sport Sciences, University of Ondokuz Mayis, 55139, Samsun, Turkey

## Abstract

This research was carried out to determine the effect of a specific single nucleotide polymorphism (SNP) region in exon 10 of the growth hormone receptor (*GHR*) gene on milk production traits in Jersey and Holstein cows raised in Turkey. Milk samples were recorded as a test day milk yield (TDMY) and an adjusted based 305 d milk yield (305-DMY). Also, milk component traits were detected. Based on the scope of this study, a total of 748 dairy cows, including 305 Holsteins raised in the Marmara Region and 163 Holstein and 280 Jersey raised in the Black Sea Region, were genotyped for the *GHR* gene using the RFLP-PCR technique. Jersey cows carrying the *GG* genotype (5.24 %) were associated with higher fat content (P<0.05). Jersey cows with *GG* and *AG* also had a higher protein content (3.44 % and 3.38 %, respectively) (P<0.05). Similarly, the protein content was the highest in Holstein cows with the *GG* genotype (3.46 %) (P<0.01), whereas Holstein cows having *AA* genotypes displayed higher TDMY (24.64 kg/d) (P<0.05) and 305-DMY (8472.4 kg) (P<0.01). The estimated increase in milk protein and fat contents due to the G allele was 0.07 % and 0.22 % in the Jersey breed, respectively. On the other hand, allele A was highly related to an increase in protein yield and 305-DMY of 0.04 and about 675 kg in the Holstein breed, respectively. The *GHR* gene should be considered as a potential candidate gene in marker-assisted selection programs to improve the performance of milk and related traits in Turkey dairy cattle populations.

## 1 Introduction

In dairy cows, new selection strategies have focused on the most important economic traits such as milk production and milk composition traits. Thus many studies have been performed to identify candidate genes associated with these types of production traits. The association test as a method is used for genetic dissection of quantitative traits based on information regarding biological, physiological, or functional processes. The variation in allelic genes in structural and regulatory regions of these genes can affect the diversification of the amount and composition of milk. Several genes have been identified in different dairy cattle breeds. One of the genes that affects these traits is the growth hormone receptor gene (*GHR*) (Hartatik et al., 2015).

The growth hormone receptor gene contains many metabolic and physiological actions and a well-known somatotropin (Rahbar et al., 2010). The growth hormone receptor gene is a member of the cytokine/hematopoietin family with three functional extracellular domains (Maj et al., 2006). The gene encodes the protein which operates as a transmembrane receptor for the growth hormone (GH). The growth hormone receptor gene mediates GH to fulfill its biological role in metabolic activity and growth on the target cell surface by transducing the signal through the cell membrane (Lincoln et al., 1995). The bovine *GHR* gene is located on chromosome 20 and contains nine (numbered 2 to 10) exonic regions in the translated part and a long 5${}^{\prime }$-noncoding region (Jiang and Lucy, 2001; Maj et al., 2004). Several genetic polymorphisms were detected in the bovine *GHR* gene. These polymorphic sites are mainly reported in the 5${}^{\prime }$-noncoding region, exon 8, and exon 10 (Aggrey et al., 1999; Blott et al., 2003; Maj et al., 2005; Viitala et al., 2006). The single nucleotide polymorphisms (SNPs) in the *GHR* gene are associated with growth performance, carcass traits, milk yield and milk composition traits, and cell differentiation. This gene affects fertility, lactogenesis, and mammary gland development in dairy cattle (Hadi et al., 2015; Maj et al., 2004; Olenski et al., 2010).

Genetic association studies are a common approach to reveal the potential relationship between the genotypes and phenotypic records, which were collected from economically important yield traits in livestock species worldwide. Unfortunately, it is challenging to find phenotypic data recorded, especially in animals that are genotyped. Thus, many studies are conducted only to determine genotypic and allele frequencies without taking phenotypic records in Turkey. For these reasons, such studies need to become widespread throughout the country to deal with shortages in this area. Determining only the polymorphic site or a prominent genetic variant generated by the causative mutation will not be enough to understand the genetic relationship. Eventually, the gene's effect on the trait of interest has to be tested statistically to reveal such an actual relationship. Such an association study was needed in which genotyping and phenotyping were performed together in dairy breeds, like Jersey and Holstein, raised in Turkey.

There were not enough studies to demonstrate the relationship between polymorphic sites of the *GHR* gene and milk-related traits in Turkish dairy populations. Therefore, using the PCR-RFLP assay, this study aimed to detect a potential association between *GHR*/*AluI* polymorphism and milk yield and milk composition traits in dairy cows raised in commercial herds.

## 2 Material and methods

The study was conducted on a total of 748 heads of Turkish dairy cattle from three different populations. Specifically, 305 of the Holstein cattle were randomly selected from a private farm in the Marmara Region, especially from among the animals that started lactation at the beginning of the study. The remaining 163 Holstein cattle and 280 Jersey cattle were randomly selected from two different private holdings in the Black Sea Region, especially from among the animals that started lactation at the beginning of the study.

The number of Jersey and Holstein cows in this study with up to five parities (from 1 to ≥5) was 30, 54, 47, 52, and 97 and 279, 137, 30, 14, and 8, respectively. Holstein is the most widely raised and well-recorded dairy animal throughout the country. On the other hand, Jersey is well adapted and grown mostly in the northern part of the country because of optimal environmental conditions for this small dairy cow. All Holstein cows were housed in free-stall barns in similar feeding conditions with free access to water sources. The animals were milked twice a day. Cows were mainly fed by total mixed ration, including alfalfa, barley grain, corn silage, corn flakes, soybean and cottonseed meals, wheat straw, sodium bicarbonate, salt, and feed additives.

Similarly, the Jersey cows were also kept in free-stall barns with open access to water sources for the whole year. However, the management feeding regime and milking were quite different compared to the Holstein herds. The Jersey cows were fed with a total mixed ration containing corn and vetch silage, concentrated feed, grass, and wheat straw. They were also able to graze for about 8–10 h on pasture during the dry season after the morning milking. The feeding management of the farms was not altered during the sampling. The Jersey cows were milked twice a day. Test day milk records and milk samples were collected once a month from 30 to 300 d of lactation and taken 10 times from each cow during the lactation periods. Some of the milk composition traits (milk protein and fat content (%), abbreviated as PC and FC, respectively) were detected with an ultrasonic milk analyzer (MilkoScan™ FT1, Foss, Hillerod, Denmark). Moreover, protein and fat yields (abbreviated as PY and FY, respectively) were calculated based on milk-related trait records. Cows with low or fairly high body condition scores (BCSs) were excluded from the study. Cows with blind quarters were excluded from the study as well.

The collection of 10 mL blood samples from an external jugular vein were collected into vacuum tubes coated with K${}_{2}$EDTA. DNA samples for molecular analyses were extracted using the standard phenol/chloroform method (Sambrook et al., 1989). DNA samples were evaluated in terms of quantity (ng/µL) and purity using the NanoDrop Spectrophotometer (Thermo Fisher Scientific Inc., USA). The PCR-RFLP method was used to genotype animals for a candidate region of the *GHR* gene (GenBank accession number: AF140284), as described in the previous study by Cobanoglu (2018). To obtain a 342 bp fragment from 158th to 499th nucleotides in exon 10 of the *GHR* gene containing a polymorphic site, the primers were designed as forward (5${}^{\prime }$-GCTAACTTCATCGTGGACAAC-3${}^{\prime }$) and reverse (5${}^{\prime }$-CTATGGCATGATTTTGTTCAG-3${}^{\prime }$) (Di Stasio et al., 2005). The thermal cycle protocol was also implemented, as mentioned in the previous study. In following PCR, the product was digested with a 10 U/µL restriction enzyme of *AluI* (Thermo Fisher Scientific Inc., USA) at 37 ${}^{\circ }$C for about 4 h to distinguish between alleles A and G. Three bands of 191, 101, and 50 bp indicated the A allele, whereas the allele G was indicated by two bands of 191 and 151 bp. DNA sequence analyses were performed to confirm the accuracy of genotyping related to the SNP region of *GHR* by purchasing the service. The confirmation of the genotyping was checked by performing double-sided DNA sequencing, both forward and reverse, for 100 samples representing all cows used in this study.

All cows were phenotyped and genotyped in terms of the traits of interest according to the daughter's design. The direct counting method was used to determine the genotypic and allelic frequencies of the *GHR* gene variant. The chi-square test ($\chi^{2}$) was performed to check if the populations were in Hardy–Weinberg equilibrium using PopGene32 (Yeh et al., 1999). The normality assumption of the data was examined with the Kolmogorov Smirnov test, and it was determined that the data were normally distributed (P>0.05). Also, the following model was used to examine the factors affecting some milk-related traits examined in the study.

$$Y_{ijklm}=\mu +\alpha_{i}+\beta_{j}+\gamma_{k}+\delta_{l}+b(X_{ijklm}-\bar{X})+e_{ijklm}$$

$Y_{ijklm}$ is the observation values, μ the population means, $\alpha_{i}$ the effect of the i genotype, $\beta_{j}$ the effect of j region (for only Holstein Friesian), $\gamma_{k}$ the effect of k lactation order, $\delta_{l}$ the effect of l calving season, b the constant regression coefficient for days in milk, $X_{ijkl}$ the ijkl subgroup, m the cow milking time, $\bar{X}$ the average milking time of the population except 305-DMY (305 d milk yield), and $e_{ijklm}$ the random error. The effect of sire was not added to the statistical model due to missing or lacking information about the sire's status. The Bonferroni test was used to determine group differences for TDMY, FC (%), FY, PC (%) and PY. However, Duncan's multiple comparison tests were used for 305-DMY. All statistical analyses were performed using IBM SPSS 21.0 (IBM Corp., 2012).

The additive (a), dominant (d), and allele substitution (α) effects of *GHR/AluI* polymorphism on milk-related traits were also calculated for both dairy breeds. The following re-parameterized equations were applied: a=(AA-GG)/2, d=AG-(AA+GG)/2, and α=a+d(q-p), where q and p represent the frequencies of alternative alleles. While the same capital letters used together represent the homozygous genotypes, the use of different letters stands for the heterozygous genotype in based on Falconer and Mackay (1996).

## 3 Results

**Figure 1:**
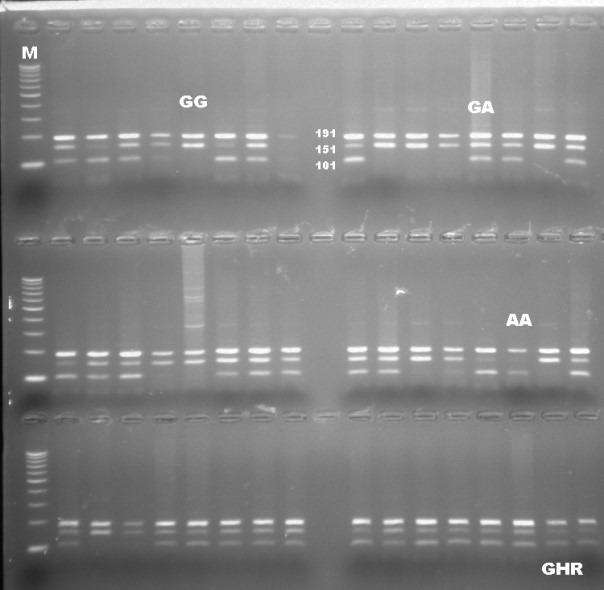
Electrophoretic separation of PCR products digested with *AluI* for the *GHR* gene.

**Figure 2:**
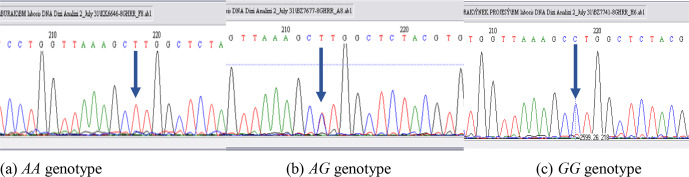
DNA sequence analyses (reverse sequences) for *AA*, *AG*, and *GG* genotypes for the *GHR* gene.

PCR-RFLP results revealed that there are three different patterns of DNA fragments existing for the *GHR* gene as the result of the digestion reaction with a restriction enzyme of *AluI*. All genotypes were precisely scored based on the banding pattern at gel electrophoresis. DNA fragments were identified as intact 191, 101, and 50 bp for *AA*, a fragment of 191, 151, 101, and 50 bp for *AG*, and 191 and 151 bp for *GG* genotypes. The DNA banding pattern in gel electrophoresis for the *GHR*/*AluI* polymorphism based on PCR-RFLP is given in Fig. 1. A part of the DNA sequence concerning the polymorphic site for *each* genotype was shown in Fig. 2. The banding results from gel electrophoresis were confirmed by DNA sequence analysis. Moreover, the A and G alleles of the *GHR* gene were identified based on the amplification of a 342 bp fragment. The result of chi-square test indicated that none of the herds were more likely to follow the Hardy–Weinberg equilibrium, due to the breeder selection criteria for milk production.

As given in Table 1, the effect of genotype on FC (P: 0.008) and PC (P: 0.002) in the present study was significantly important, while TDMY, 305-DMY, FY, and PY were not affected by genotype in Jersey cows. The effect of parity and calving season on milk yield and milk components in Jersey cows has already been reported in a previous study (Kul et al., 2018). According to the results, there is a significant effect of parity on TDMY, 305-DMY, PC, and PY, but FC and FY did not differ significantly among the parity groups. Also, neither the milk yield nor the milk components were significantly affected by the calving season. Genotype had a significant effect on TDMY (P: 0.004), 305-DMY (P: 0.004), FY (P: 0.092), PC (P: 0.010), and PY (P: 0.037) in Holstein cows, but no significant effect on FC was observed. Parity had an important effect on FY (P: 0.046), while TDMY, 305-DMY, FC, PC, and PY were not affected by parity. Overall, none of the milk yield and milk component traits were significantly influenced by the calving seasons. However, the effect of the herd on TDMY, 305-DMY, FC, FY, and PY (P<0.001), except for PC, was also statistically significant in Holstein cows.

**Table 1:** Variance analysis for milk yield and milk components according to genotype, parity, calving season and herd.

Breeds	Factors	TDMY (kg/d)	305-DMY (kg)	FC (%)	${}^{*}$FY (kg/d)	PC (%)	${}^{*}$PY (kg/d)
Jersey	Genotype	0.117	0.152	0.008	0.682	0.002	0.263
	Parity	0.013	0.001	0.349	0.069	0.028	0.037
	Calving season	0.266	0.315	0.668	0.930	0.957	0.265
	b	-0.009	–	0.002	< 0.001	< 0.001	< 0.001
	p	< 0.001	–	0.001	0.158	0.039	< 0.001
Holstein	Genotype	0.004	0.004	0.198	0.092	0.010	0.037
	Parity	0.255	0.888	0.650	0.046	0.338	0.346
	Calving season	0.338	0.192	0.833	0.294	0.239	0.415
	Herd	< 0.001	< 0.001	< 0.001	< 0.001	0.164	< 0.001
	b	-0.006	–	< 0.001	< 0.001	< 0.001	< 0.001
	p	0.009	–	0.889	0.009	0.020	0.068

TDMY: test day milk yield; 305-DMY: 305 d milk yield; FC: milk fat content; FY: fat yield; PC: milk protein content; PY: protein yield. ${}^{*}$ FY and PY (kg/d) were calculated based on TDMY. b: correction coefficients; p: significance.

The means and standard deviation values (SD) for milk production and milk composition traits are given in Table 2 based on *GHR*/*AluI* genotyping for the Jersey breed raised in the Black Sea Region. The statistical analysis has shown that there was a significant difference for test day FC and PC (P<0.05), but TDMY, 305-DMY, test day FY, and PY were similar to each other among Jersey cows based on three genotypic groups. The highest FC (5.24 %) was observed in Jersey cows with the *GG* genotype, but the lowest one was in animals with *AA* (4.80 %) and *AG* genotypes (4.97 %) (P<0.05). On the other hand, cows with *GG* (3.44 %) and *AG* genotypes (3.38 %) had a higher PC than cows with the *AA* genotype (3.30 %).

**Table 2:** Comparison results for the milk-related traits examined in Jersey cattle based on *GHR*/*AluI* genotypes (mean ± SD).

Genotype	n	TDMY (kg/d)	305-DMY (kg)	FC (%)	${}^{*}$FY (kg/d)	PC (%)	${}^{*}$PY (kg/d)
*AA*	22	16.18 ± 3.6	5155.3 ± 1206.5	4.80 ± 0.4${}^{b}$	0.78 ± 0.18	3.30 ± 0.10${}^{b}$	0.50 ± 0.11
*AG*	204	16.03 ± 3.3	5166.1 ± 1408.1	4.97 ± 0.6${}^{b}$	0.79 ± 0.16	3.38 ± 0.16${}^{a}$	0.54 ± 0.11
*GG*	54	14.96 ± 3.1	4873.9 ± 1301.7	5.24 ± 0.7${}^{a}$	0.77 ± 0.14	3.44 ± 0.20${}^{a}$	0.51 ± 0.10

${}^{a,\;b}$ Different exponents in a column indicate a significant difference with P < 0.05. TDMY: test day milk yield; 305-DMY: 305 d milk yield; FC: milk fat content; FY: fat yield; PC: milk protein content; PY: protein yield. ${}^{*}$ FY and PY (kg/d) were calculated based on TDMY.

The same parameters for all milk-related traits for *GHR*/*AluI* polymorphism are given in Table 3 for all Holstein cows raised in the Black Sea and Marmara regions. There were also significant differences between the three genotypic groups of Holstein cows for some of the milk traits. Holstein cows were significantly different for TDMY (P<0.05) and 305-DMY and milk PC at P<0.01. The Holstein cows bearing the *AA* genotype were observed to be importantly higher for TDMY (24.64 kg/d) than animals carrying the *GG* genotype (19.40 kg). Similarly, cows with *AA* were significantly higher (8472.4 kg) than animals with the *GG* genotype (7032 kg) for 305-DMY.

**Table 3:** Comparison results for the milk-related traits examined in Holstein cattle based on *GHR*/*AluI* genotypes (mean ± SD).

Genotype	n	TDMY (kg/d)	305-DMY (kg)	FC (%)	${}^{*}$FY (kg/d)	PC (%)	${}^{*}$PY (kg/d)
*AA*	239	24.64 ± 6.39${}^{a}$	8472.4 ± 2238.9${}^{A}$	3.78 ± 0.61	0.91 ± 0.22	3.16 ± 0.27${}^{B}$	0.77 ± 0.19
*AG*	223	23.45 ± 6.13${}^{\mathrm{ab}}$	7842.7 ± 2170.8${}^{\mathrm{AB}}$	3.87 ± 0.61	0.89 ± 0.22	3.21 ± 0.25${}^{B}$	0.74 ± 0.18
*GG*	6	19.40 ± 5.29${}^{b}$	7032.0 ± 1854.7${}^{B}$	4.04 ± 0.55	0.78 ± 0.24	3.46 ± 0.28${}^{A}$	0.67 ± 0.20

${}^{a,\;b}$ Different exponents within a column indicate a significant difference with P < 0.05. ${}^{A,\;B}$ Different superscripts within a column indicate significant difference with P < 0.01. TDMY: test day milk yield; 305-DMY: 305 d milk yield; FC: milk fat content; FY: fat yield; PC: milk protein content; PY: protein yield. ${}^{*}$ FY and PY (kg/d) were calculated based on TDMY.

By contrast, Holstein cows with the *GG* genotype were significantly higher (3.46 %) than animals with *AA* and *AG* genotypes with 3.78 % and 3.87 %, respectively, for test day PC within whole milk. In contrast, the differences between all other traits (FC, FY, and PY) were not significantly important. Yet, animals with the *AA* genotype showed higher values for all of these essential yield traits than cows with *AG* and *GG* genotypes. Overall, the associations of *GHR*/*AluI* with FC and PC were revealed to be significant in Jersey cows at P<0.05. On the other hand, TDMY at P<0.05, 305-DMY, and PC at P<0.01 were significantly related to *GHR*/*AluI* polymorphism in Holstein cows. Specifically, it is worth noting that animals carrying *AA* and *AG* genotypes were associated with higher TDMY and 305-DMY but a lower PC than the animals having the *GG* genotype.

The estimated effects of *GHR*/*AluI* on milk production traits in Jersey and Holstein cows are shown in Tables 4 and 5, respectively. Additive and dominant gene actions and allele substitution effects were tested in two different dairy breeds for a given gene locus. Significant additive effects of *GHR* on FC and PC (P<0.05) and dominant effects on TDMY, 305-DMY, and PY (P<0.05) were observed in the Jersey herd. On the other hand, highly important additive effects for 305-DMY (P<0.05), FC, and PY (P<0.05) and dominant effects for TDMY (P<0.05), PC (P<0.05), and FY (P<0.01) were detected in Holstein herds. For both breeds, it is worth noting that the allele A of this marker was significantly associated with TDMY, 305-DMY, and PY, whereas the allele G was highly associated with FC and PC. The estimated increase in milk FC and PC of the G allele was 0.07 % and 0.22 % compared to the allele A in the Jersey breed, respectively. At the same time as the favorable allele, the allele A was highly related to an increase in PY and 305-DMY of 0.04 kg/d and about 675 kg, respectively, compared with the allele *G* at this marker in the Holstein breed.

**Table 4:** Genetic effects of *GHR*/*AluI* polymorphism on milk-related traits in the Jersey breed.

Locus	Traits	Additive	Dominant	Allele substitution
		effect (a)	effect (d)	effect (α)
*GHR*/*AluI*	TDMY (kg/d)	0.61	0.46${}^{a}$	0.6652
	305-DMY (kg)	140	151.5${}^{a}$	158.88
	FC %	$-0.22^{a}$	-0.05	$-0.226^{a}$
	${}^{*}$FY (kg/d)	0.005	0.015${}^{a}$	0.0068
	PC %	$-0.07^{a}$	0.01	$-0.0688^{a}$
	${}^{*}$PY (kg/d)	-0.005	0.035${}^{a}$	-0.0008

${}^{a}$ Significant at P < 0.05. TDMY: test day milk yield; 305-DMY: 305 d milk yield; FC: milk fat content; FY: fat yield; PC: milk protein content; PY: protein yield. ${}^{*}$ FY and PY (kg/d) were calculated based on TDMY.

**Table 5:** Genetic effects of *GHR*/*AluI* polymorphism on milk-related traits in Holstein breed.

Locus	Traits	Additive	Dominant	Allele substitution
		effect (a)	effect (d)	effect (α)
*GHR*/*AluI*	TDMY (kg/d)	2.62	1.43${}^{a}$	1.905
	305-DMY (kg)	720.2${}^{b}$	90.5	674.95${}^{b}$
	FC %	$-0.13^{a}$	-0.04	$-0.11^{a}$
	${}^{*}$FY (kg/d)	0.065	0.045${}^{b}$	0.0425
	PC %	-0.15	$-0.1^{a}$	-0.1
	${}^{*}$PY (kg/d)	0.05${}^{a}$	0.02	0.04${}^{a}$

${}^{a}$ Significant at P < 0.05. ${}^{b}$ Significant at P < 0.01. TDMY: test day milk yield; 305-DMY: 305 d milk yield; FC: milk fat content; FY: fat yield; PC: milk protein content; PY: protein yield. ${}^{*}$ FY and PY (kg/d) were calculated based on TDMY.

## 4 Discussion

Many studies have been conducted to detect the effect of genetic polymorphism of *GHR* on milk yield and related traits in different cow breeds (Dario et al., 2008; Komisarek et al., 2011; Li-Juan et al., 2009; Kiyici et al., 2019). In this study, we described the use of the SNP marker identified in the *GHR* gene in two dairy cow breeds – Jersey and Holstein cows – to determine if this marker might be used for selection purposes to improve milk yield and milk quality levels in these populations.

According to the least square analyses in Jersey cows, there were significant relationships between the *GHR*/*AluI* polymorphism and FC and PC (P<0.05) but no association had been found with TDMY, 305-DMY, FY, and PY in the same breed. Jersey cows with the *GG* genotype had a higher FC than cows with *AA* and *AG* genotypes. Moreover, PC was higher in cows with the *AG* and *GG* genotypes than the ones with the *AA* genotype. However, the TDMY and 305-DMY were higher in cows with *AA* and *AG* genotypes in comparison with the *GG* genotype, but they were non-significant. Therefore, based on the available findings, this SNP polymorphism detected only in *GHR* would not be expected to increase significantly in marker-assisted selection for milk yield, fat, and protein yield traits in Jersey cows. Therefore, it would be necessary to employ other polymorphic loci that have a statistically greater effect in increasing the activity of this candidate gene. On the other hand, the results of the present study are in agreement with previous observations achieved by Dario et al. (2008), who reported that *GHR* causes a significant increase in the milk yield performance of Jersey cows. In general, when evaluating the causes of different results in Jersey cows, the geographical and environmental differences in which studies are conducted, variations in the genetic background of the sires, and differences between the genetic regions genotyped in the studies must be taken into account to make an accurate and unbiased assessment.

In contrast to the present study, Komisarek et al. (2011) reported a strong relationship between the SNP marker and milk yield, FY, and PY. The findings may be assessed as the SNP locus in the *GHR* gene was not polymorphic enough to detect a strong association for milk yield traits in Jersey cows. Thus, the different regions in the *GHR* gene might be associated with the milk yield and its components in Jersey cows.

The impact of *GHR*/*AluI* polymorphism on TDMY, 305-DMY, and PC in Holstein cows was significant in the current study. However, FC and FY do not appear to be significantly affected by the different genotypes. One of the reasons for this may be that herd size is not sufficient to reach statistical power to determine the genotypic effect on milk yield and protein performance. However, the effect of genes that are effective regarding traits with economic importance is evaluated by haplotype analysis, and the expected progress can be achieved through molecular genetic breeding. Despite this, some authors (Aggrey et al., 1999; Banos et al., 2008; Li-Juan et al., 2009; Kiyici et al., 2019) observed that *GHR*/*AluI* polymorphisms had a significant effect on milk yield traits in Holstein cows. The results were also in the study conducted by El-Nahas et al. (2018), who stated that there was a significant relationship between *GHR*/*AluI* and PC. By contrast, in a study about a gene related to *GHR*, Hartatik et al. (2015) determined the association between *GH* gene polymorphism on FC (%) in Friesian Holstein cow groups from New Zealand and Australia. The results obtained in the current study were different from the other studies conducted by Lechniak et al. (2002) and Arslan et al. (2016), who found that no statistically significant difference was determined between *GHR* gene polymorphism and lactation and daily milk yields. As shown in Table 1, the reasons for the differences in the quantitative characteristics of Holstein cows raised in two different herds can be attributed to the fact that they were raised in different regional conditions of Turkey and have different management feeding regimes and different environmental conditions. Moreover, El-Nahas et al. (2018) stated that the impact of *GH *genes on 305-DMY and FC in Holstein cows was not significantly important. Kiyici et al. (2019) reported that no significant relationships were observed between *GH*/*TaqI *polymorphism and milk fat and protein content. However, Li-Juan et al. (2009) detected significant associations between *GHR* genotypes and milk performance traits in Chinese Holstein cows, as outlined in this current study. The inconsistencies among the research results are mostly due to the differences in SNP marker locations, breed differences, the number of animals employed in this research, unbalanced genotypes between cows, and the reliability of collected milk data.

The growth hormone and its receptor gene polymorphisms and their associations with milk yield and milk composition in other cattle breeds have also been documented in many reports. In the current study, we reported significantly higher TDMY and 305-DMY in cows with the *AA* genotype, but the lowest in cows with the *GG* genotype in Holstein breed. Similar conclusions were consistent with the results presented by Lucy et al. (1993), who reported that Holstein cows with the *LL* genotype of *GH* release more milk than the *VV* genotypes. Similarly, Dybus (2002) reported that cows with the *LL* genotype at the *GH* gene had a higher milk yield and PY in German Black-and-White cattle. Grochowska et al. (2001) determined that the milk yield in Simmental cattle was positively related to the *LV* genotype at the *GH*/*AluI *site. Moreover, El-Nahas et al. (2018) reported that *GHR/Msp1* was associated with the different milk composition characteristics in Baladi cattle. In a recent study, Kiyici et al. (2019) also reported that the cows with the *LL* genotype had higher milk yields than Turkish Holstein cows with the *LV* genotype in agreement with our findings. However, Khatami et al. (2005) reported that *GH1*/*AluI* polymorphisms were not associated with milk yield traits in the Holstein and Yaroslavl breeds.

We also reported the results about milk component traits in which cows carrying the *GG* genotype had the highest PC than those of alternative genotype carriers. However, no significant relationships were observed between *GHR*/*AluI* and FC, FY, and PY in Holstein cows. Viitala et al. (2006) concluded somewhat similar results in different SNP polymorphic sites at the *GHR* gene that *GHR*/*F279Y* had the most significant influence on PC and FC in Finnish Ayrshire cows. Contrary to the current results, Li-Juan et al. (2009) reported that cows carrying the *TT* genotype had a higher FC than Holstein cows having *AA* at different polymorphic sites of the *GHR* gene. One of the studies conducted with indigenous cattle breeds of Turkey, Yardibi et al. (2009) found that both South Anatolian and East Anatolian Red cattle in Turkey with the *VV* genotype in the *GH* gene had a higher FC than other genotypes, which is verified in our results in the Jersey breed. Although Holstein and Jersey cows are both dairy animals, one of the main reasons why the detected SNP marker, which has an effect on some milk-yield-related traits in one breed, may not have the same type of effect in the other breed is that there are some fundamental differences in production performance between them in terms of general breed characteristics. Furthermore, the difference between results from Holsteins and Jerseys could also be the result of a linkage disequilibrium between *GHR*/*AluI* and the real causative mutation. In other words, the *GHR*/*AluI* could be linked to the real causative mutation but in a different phase between breeds. General reasons for the observed differences among the studies about the effect of *GHR* on milk-yield-related traits are mainly the different allele frequencies of the animals in the herds, differences in the applied statistical model, genetic backgrounds of animals, and differences between environmental factors. For these reasons, further studies are needed to employ more cow breeds and many more animals within breeds raised in different conditions in Turkey. Taken together, appropriate studies on the association of SNP markers at GH and its receptor genes in different cattle breeds prove that there are potential polymorphic regions in these genes which substantially affect the variations in milk production and milk component traits.

## 5 Conclusion

The present study demonstrated that the SNP polymorphism of the *GHR* gene had a great effect on fat content and protein content in the Jersey population. Based on our findings, there was a strong probability of associations between the *GG* genotype with fat content and also *GG* and *AG* genotypes with protein content in Jersey cows. The association between the *AA* genotype with milk yield traits in Holstein cows was more robust than the *GG* genotype. Also, we presented a strong association between *GG* genotypes and protein content of milk among Holstein cows. Thus, the current results may provide a novel aspect for evaluating probable genetic markers in this genomic region. But it has yet to be checked if the detected SNP marker in the *GHR* gene is the actual causative mutation for milk-yield-related traits or whether the SNP location is in linkage disequilibrium with other candidate genes for production traits in further studies. Consequently, it may be suggested that the *GHR*/*AluI* variants should be considered as markers in molecular-based selection programs to improve the amount and the ratio of milk-related traits in the dairy cattle populations of Turkey.

## Data Availability

The data sets are available upon request from the corresponding author.

## References

[bib1.bib1] Aggrey S, Yao J, Sabour M, Lin C, Zadworny D, Hayes J, Kuhnlein U (1999). Markers within the regulatory region of the growth hormone receptor gene and their association with milk-related traits in Holsteins. J Hered.

[bib1.bib2] Arslan K, Taheri S, Sener EF, Akuz B, Akcay A, Ozkül Y, Iscan KM (2016). Investigation of the promoter polymorphisms of the growth hormone (GH1), growth hormone receptor (GHR), insulin-like growth factor (IGF-I), and prolactin (PRL) genes and the correlation between gene expression and milk yields in Holstein cattle raised in Central Anatolia. Turkish Journal of Veterinary and Animal Sciences.

[bib1.bib3] Banos G, Woolliams JA, Woodward BW, Forbes AB, Coffey MP (2008). Impact of single nucleotide polymorphisms in leptin, leptin receptor, growth hormone receptor, and diacylglycerol acyltransferase (DGAT1) gene loci on milk production, feed, and body energy traits of UK dairy cows. J Dairy Sci.

[bib1.bib4] Blott S, Kim J-J, Moisio S, Schmidt-Küntzel A, Cornet A, Berzi P, Cambisano N, Ford C, Grisart B, Johnson D, Karim L, Simon P, Snell R, Spelman R, Wong J, Vilkki J, Georges M, Farnir F, Coppieters W (2003). Molecular dissection of a quantitative trait locus: A phenylalanine-to-tyrosine substitution in the transmembrane domain of the bovine growth hormone receptor is associated with a major effect on milk yield and composition. Genetics.

[bib1.bib5] Cobanoglu O (2018). Genetic diversity in terms of GHR gene in some cattle breeds raised in Turkey. Turkish Journal of Agriculture-Food Science and Technology.

[bib1.bib6] Dario C, Carnicella D, Ciotola F, Peretti V, Bufano G (2008). Polymorphism of growth hormone GH1-AluI in Jersey cows and its effect on milk yield and composition. Asian-Australas J Anim Sci.

[bib1.bib7] Di Stasio L, Destefanis G, Brugiapaglia A, Albera A, Rolando A (2005). Polymorphism of the GHR gene in cattle and relationships with meatproduction and quality. Anim Genet.

[bib1.bib8] Dybus A (2002). Associations between Leu/Val polymorphism of growth hormone gene and milk production traits in Black-and-White cattle. Arch Tierzucht.

[bib1.bib9] El-Nahas A, Basiony W, El-Kassas S, Mahmoud S (2018). Variation in the genetic effects of ABCG2, growth hormone and growth hormone receptor gene polymorphisms on milk production traits in Egyptian Native, Holstein and hybrid cattle populations. Pak Vet J.

[bib1.bib10] Falconer D, Mackay T (1996). Introduction to quantitative genetics.

[bib1.bib11] Grochowska R, Sørensen P, Zwierzchowski L, Snochowski M, Løvendahl P (2001). Genetic variation in stimulated GH release and in IGF-I of young dairy cattle and their associations with the leucine/valine polymorphism in the GH gene. J Anim Sci.

[bib1.bib12] Hadi Z, Atashi H, Dadpasand M, Derakhshandeh A, Ghahramani Seno MM (2015). The relationship between growth hormone polymorphism and growth hormone receptor genes with milk yield and reproductive performance in Holstein dairy cows. Iran J Vet Res.

[bib1.bib13] Hartatik T, Kurniawati D, Adiarto A (2015). Associations between polymorphism of growth hormone gene with milk production, fat and protein content in Friesian Holstein cattle. J Indones Trop Anim Agric.

[bib1.bib14] IBM Corp (2012). IBM SPSS Statistics for Windows, Version 21.0, Armonk.

[bib1.bib15] Jiang H, Lucy MC (2001). Variants of the 5${}^{\prime }$-untranslated region of the bovine growth hormone receptor mRNA: isolation, expression and effects on translational efficiency. Gene.

[bib1.bib16] Khatami SR, Lazebny OE, Maksimenko VF, Sulimova GE (2005). Association of DNA polymorphisms of the growth hormone and prolactin genes with milk productivity in Yaroslavl and Black-and-White cattle. Russ J Genet.

[bib1.bib17] Kiyici JM, Arslan K, Akyuz B, Kaliber M, Aksel EG, Cinar MU (2019). Relationships between polymorphisms of growth hormone, leptin and myogenic factor 5 genes with some milk yield traits in Holstein dairy cows. Int J Dairy Technol.

[bib1.bib18] Komisarek J, Michalak A, Walendowska A (2011). The effects of polymorphisms in DGAT 1, GH and GHR genes on reproduction and production traits in Jersey cows. Anim Sci P.

[bib1.bib19] Kul E, Abacı SH, Çobanoğlu Ö, Gürcan EK, Çankaya S (2018). Estimation of genetic and environmental parameters for milk traits in jersey cows. Mediterranean Agricultural Sciences.

[bib1.bib20] Lechniak D, Strabel T, Przybyła D, Machnik G, Świtoński M (2002). GH and CSN3 gene polymorphisms and their impact on milk traits in cattle. J Anim Feed Sci.

[bib1.bib21] Li-Juan W, Qiu-Ling L, Chang-Fa W, Hong-Mei W, Jian-Bin L, Yun-Dong G, Ming-Hai H, Ji-Feng Z (2009). CRS-PCR polymorphisms of the GHR gene and its relationship with milk production traits in Chinese Holstein cows. Chinese Journal of Agricultural Biotechnology.

[bib1.bib22] Lincoln DT, Sinowatz F, El-Hifnawi E, Hughes RL, Waters M (1995). Evidence of a Direct Role for Growth Hormone (GH) in Mammary Gland Proliferation and Lactation. Anatomia, Histologia, Embryologia.

[bib1.bib23] Lucy MC, Hauser SD, Eppard PJ, Krivi GG, Clark JH, Bauman DE, Collier RJ (1993). Variants of somatotropin in cattle: Gene frequencies in major dairy breeds and associated milk production. Domest Anim Endocrinol.

[bib1.bib24] Maj A, Oprzadek J, Oprzadek A, Dymnicki E, Zwierzchowski L (2004). Polymorphism in the 5${}^{\prime }$-noncoding region of the bovine growth hormone receptor gene and its association with meat production traits in cattle. Anim Res.

[bib1.bib25] Maj A, Pareek CS, Klauzińska M, Zwierzchowski L (2005). Polymorphism of 5${}^{\prime }$-region of the bovine growth hormone receptor gene. J Anim Breed Genet.

[bib1.bib26] Maj A, Oprządek J, Dymnicki E, Zwierzchowski L (2006). Association of the polymorphism in the 5′-noncoding region of the bovine growth hormone receptor gene with meat production traits in Polish Black-and-White cattle. Meat Science.

[bib1.bib27] Olenski K, Suchocki T, Kaminski S (2010). Inconsistency of associations between growth hormone receptor gene polymorphism and milk performance traits in Polish Holstein-Friesian cows and bulls. Anim Sci P.

[bib1.bib28] Rahbar R, Rahimi G, Pirsaraei ZA, Gholizadeh M (2010). Identification of polymorphism in promoter region of growth hormone receptor (GHR) gene and its association with milk related traits in Holstein cows. Afr J Biotechnol.

[bib1.bib29] Sambrook J, Fritsch EF, Maniatis T (1989). Molecular cloning: a laboratory manual.

[bib1.bib30] Viitala S, Szyda J, Blott S, Schulman N, Lidauer M, Mäki-Tanila A, Georges M, Vilkki J (2006). The Role of the bovine growth hormone receptor and prolactin receptor genes in milk, fat and protein production in Finnish Ayrshire dairy cattle. Genetics.

[bib1.bib31] Yardibi H, Hosturk GT, Paya I, Kaygisiz F, Ciftioglu G, Mengi A, Oztabak K (2009). Associations of growth hormone gene polymorphisms with milk production traits in South Anatolian and East Anatolian Red cattle. J Anim Vet Adv.

[bib1.bib32] Yeh F, Yang R, Boyle T (1999). Popgene. Microsoft windows-based freeware for population genetic analysis, Release 1.31.

